# Impact on Health, Resources, and Satisfaction: A Qualitative Study of Primary Health Care Case-Management Nurses

**DOI:** 10.3390/healthcare13090974

**Published:** 2025-04-23

**Authors:** María José Molina-Gil, María Dolores Guerra-Martín, Rocío De Diego-Cordero

**Affiliations:** 1 South Seville Health Management Area, Andalusian Health Service, 41071 Seville, Spain; mariajmg@euosuna.org; 2 Nursing School, Osuna University, 41640 Seville, Spain; 3 Nursing Department, Faculty of Nursing, Physiotherapy and Podiatry, University of Seville, 41009 Seville, Spain; rdediego2@us.es; 4 Institute of Biomedicine of Seville (IBiS), 41013 Seville, Spain

**Keywords:** case managers, chronic disease, primary care nursing, qualitative research

## Abstract

The aging population and the increasing prevalence of chronic diseases necessitate new healthcare models. Case-Management Nurses (CMNs) emerge as a promising alternative to enhance patient care. Objective: To explore CMNs’ perceptions of the impact on health, resources, and professional satisfaction. Methods: A qualitative study using semi-structured interviews was conducted with CMNs from a southern Spanish province. This study adheres to the Standards for Reporting Qualitative Research (SRQR). The population consisted of 61 CMNs. Three analytical categories of a theoretical or deductive nature were identified, directly related to this study’s objective. This study was approved by the Andalusian Biomedical Research Ethics Committee (Code: 1139-N-22) and conducted in accordance with the Declaration of Helsinki. Data analysis was performed using ATLAS.ti. Results: The sample was comprised of 31 CMNs (24 women). The mean age was 56.3 years. A total of 12 CMNs had more than 20 years of experience. Interviews were conducted between October and November 2022. Within each category, different emerging subcategories were identified: 1. Impact on health: Patients and caregivers. 2. Impact on resources: Computer tools, effectiveness/efficiency of the CMNs, and material resources. 3. Impact on professional satisfaction: Positive professional satisfaction and negative professional satisfaction. Conclusions: The findings suggest that the practice of CMNs in primary care improves the health and quality of life of patients and their caregivers while reducing healthcare resource utilization. CMNs reported high levels of job satisfaction. These findings support the implementation of this care model to optimize the management of chronic patients in home and residential care settings. However, larger-scale quantitative studies are needed to confirm these results and explore their generalizability.

## 1. Introduction

Demographic indicators show approximately 13.4% of the world’s 8.86 billion individuals are over 60 years old. They also point to foreseen 16.5% and 30% increases by 2030 and 2060, respectively. These data signal that the proportion of the population over 60 is much larger than in the past, as life expectancy is at least 60 years old in most people [1]. By 2050, the world population of individuals aged over 60 years old will almost have doubled, rising from 12% to 22%; in other words: 2.1 billion people. It is expected that the number of people aged at least 80 years old will have tripled by 2050, reaching approximately 426 million individuals. Given all of the aforementioned information, it is necessary to promote strategies that allow for healthy aging [2].

Population aging and longer life expectancy are causing an increase in the number of people affected by chronic diseases. New care models that incorporate intermediate assistance are required, focusing on comprehensive, multidisciplinary, and interlevel assistance, thus improving communication between in-hospital care and Primary Health Care (PHC) [3,4].

Chronic diseases are gradually increasing in terms of prevalence and causing more activity in PHC; consequently, new healthcare models are required, which should include advanced practice profiles. Such is the case of Case-Management Nurses (CMNs), a career implemented in Andalusia (Spain) since 2002 that is considered as advanced practice nursing. The World Health Organisation (WHO) and the Pan-American Health Organisation (PAHO) are promoting the implementation of Advanced Practice Nursing (especially in the PHC scope) to improve access to health services and comprehensive care [5,6,7,8,9,10,11].

The assistance provided by CMNs in PHC supposes a 63.76% reduction in the number of admissions when compared to habitual care, which only reduces it by 31.60%. In relation to savings, the calculation supposes a minimum of EUR 1,165,164.36 per year, preventing 1938 hospitalisation days. Therefore, new care models that incorporate this figure in PHC are required, in addition to consequently adapting to the new needs given this increase in the prevalence of chronic diseases that demand special assistance, especially from the home environment, fostering healthy lifestyles and empowering patients to improve their quality of life. In total, 60% of the hospital admissions can be prevented through intervention strategies via PHC, including duly trained and qualified nurses, as is the case of CMNs. Post-hospital discharge follow-up in charge of nursing personnel reduces readmission risk. Telenursing improves access to healthcare, optimizing resources and enhancing patients’ quality of life in PHC [12,13,14,15].

CMNs is concerned with accompanying the patient and family through the healthcare system, applying actions that are effective for the patient and efficient for the healthcare system. CMNs focuses its activity on identifying populations requiring com-plex care, including patients requiring palliative care in home settings, for which continu-ity of care and interprofessional coordination are essential to ensure compliance with the therapeutic plan [6,8,16,17,18].

The impact on satisfaction is evident in numerous studies, showing a decrease in the use of emergency services, hospital admissions, and readmissions, which improves the satisfaction of patients with chronic diseases including diabetes, hypertension, chronic obstructive pulmonary disease, and cancer due to interventions in consultations, home visits, and telephone contacts. Family caregivers also express this satisfaction, as they receive emotional and instrumental support as well as active listening. In order to demonstrate this satisfaction, a model is needed that integrates these practices at an international level, including protocols and evidence-based clinical practice guidelines, reducing variability [11,17].

CMNs incorporate a quality management system, obtaining high levels of satisfaction and meeting the needs and expectations of citizens and professionals, since they promote excellent and quality practice. The objective of CMNs is the satisfaction of patients, families, citizens in general, and professionals. Case management was an important innovation in home care, which increased the satisfaction of patients and family caregivers, reducing the level of overload [16,18].

The interventions implemented by CMNs also mitigate psychological distress in patients and caregivers alike, in addition to providing improvements in relation to patient safety, thus helping to improve their quality of life and fostering self-management and personal accountability. It is also deemed necessary to integrate digital technologies, as they would provide better efficacy in health institutions, acting as a resource for professionals and patients alike and improving all managerial activities. This translates to high satisfaction levels regarding CMNs due to patients and caregivers acknowledging their work, the emotional and instrumental support provided, and the accessibility they offer, both in-person and via telephone calls [17,19,20,21].

As for PHC teams, CMNs assist patients who require special care due to their complex conditions, such as those in need of palliative care and their caregivers. This provides them with great satisfaction because they feel like key reference figures, despite their ever increasing workload caused by sociodemographic changes [8,18,22,23].

CMNs express satisfaction with their professional activity, but they need to better define their roles—as there is a lack of deep understanding of the subjective experiences of the roles of these professionals—so that this professional profile is better known [24]. While quantitative data suggest a positive impact on resource utilization, the perceptions of CMNs regarding their contribution to this efficiency warrant further exploration.

The aim of this study is to explore CMNs’ perceptions of their impact on health, resources, and professional satisfaction.

## 2. Materials and Methods

### 2.1. Research Design

A qualitative, exploratory, and observational study was conducted using a phenomenological approach to understand the meaning of an experience by identifying themes and sub-themes emerging from participant observation and discourse. Guided by a phenomenological–hermeneutic paradigm, following Amadeo Giorgi’s theoretical framework [25], this study employed qualitative methods through interviews based on the pre-established script. This qualitative method provides researchers with substantial flexibility, allowing them to gain richer insights into participants’ views. It also supports diverse interpretations of respondents’ opinions on a subject and can reveal aspects that might be missed when using survey-based approaches. Anchored in a phenomenological approach, this study aimed to explore and interpret central themes emerging from participants’ lived experiences.

### 2.2. Setting and Sampling

A mix of convenience sampling and snowball sampling techniques was used to recruit participants according to specific eligibility requirements. The main requirement was having direct experience with the phenomenon being studied. The researchers aimed to include individuals who shared that experience but varied in their backgrounds and viewpoints, to enrich the findings and align with this study’s goals. Eligible participants were CMNs working in Health Districts (HDs) from the province of Seville. Excluded from the study were CMNs working outside of Health Districts (HDs) from the province of Seville. The figure of the CMNs was introduced in the province of Seville (Spain) in 2002, based on Decree 137/2002 on Support for Andalusian Families [8].

From a total of 61 active CMNs at the time of this study, 31 PHC CMNs working at the five HDs from the province of Seville were selected: East Seville HD (ESHD) with a population of 5 and a sample of 4, South Seville HD (SSHD) with a population of 13 and a sample of 7, Seville HD (SHD) with a population of 25 and a sample of 13, Aljarafe HD (AHD) with a population of 9 and a sample of 4, and North Seville HD (AHD) with a population of 9 and a sample of 3.

### 2.3. Data Collection

Semi-structured interviews were conducted to explore the participants’ experiences, focusing on uncovering main themes and subthemes through their discourse and observational data. Data were collected in 2024. Each interview, conducted in Spanish, lasted between 50 and 60 min and continued until data saturation was achieved.

Face-to-face interviews were typically held in staff break rooms during work pauses, often over coffee to foster a more comfortable setting.

Three researchers participated in this study [M.J.M.-G., M.D.G.-M., and R.d.D.-C.], two of them with experience in qualitative research [M.D.G.-M. and R.d.D.-C.]. One of the researchers was in charge of conducting the interviews [M.J.M.-G.]. Key informants in each Primary Care District were contacted through the Primary Care District Directorates to schedule the interviews.

The CMNs that took part in this study were duly informed about its details and provided their consent in writing. After due authorization, the interviews were recorded, transcribed, coded, and grouped into three categories and seven subcategories. The interviews were transcribed manually. No software was used. The transcripts were not sent to the informants for validation.

### 2.4. Data Analysis

Thematic analysis was carried out using a combination of inductive and deductive approaches, following these steps: (1) data familiarization, (2) initial categorization, (3–5) identification, review, and refinement of themes, and (6) compilation of the final report, which included participant quotes labeled by letter, gender, and age. ATLAS.ti 24 was used for data analysis, allowing for segmenting the verbatim statements and easing coding. The Standards for Reporting Qualitative Research (SRQR) [26] were followed.

The three analysis categories identified in this study (1. Impact on health, 2. Impact on resources, and 3. Impact on professional satisfaction) were theoretical or deductive. They were defined a priori, directly in relation to the primary research aim, which was to explore the perceptions of CMNs regarding these three specific aspects. While the main categories were theoretically defined based on the study objectives, the specific themes that emerged within each category (subcategories) were identified emergently through the detailed analysis of the data. Information saturation was achieved through the iterative process of identifying and refining themes.

The qualitative data analysis was conducted by two researchers [M.J.M.-G., R.d.D.-C]. Initially, each researcher independently analyzed the dataset. Subsequently, investigator triangulation was performed to enhance the credibility and validity of the findings. During this process, the two researchers analyzed and compared their interpretations and coding of the data. Discrepancies and convergences in their analyses were discussed to achieve a richer and more consensual understanding of the results. In instances where significant discrepancies in data interpretation arose, a third researcher [M.D.G.-M.] acted as an arbiter.

### 2.5. Ethical Consideration

This study was approved by the Andalusian Biomedical Research Ethics Committee (Spain) (Code: 1139-N-22) and was conducted according to the Declaration of Helsinki.

All participants gave their written informed consent, and all data were anonymized to protect participant confidentiality.

### 2.6. Trustworthiness

In qualitative research, trustworthiness encompasses the credibility, transferability, confirmability, and dependability of the results. Credibility refers to how accurate and genuine the data and findings are. To ensure this, member checking was used, where participants reviewed and confirmed the findings. The researchers also spent extended time with the data to deeply understand the participants’ perspectives. Triangulation, by comparing different interviews and observations, further strengthened the trustworthiness of the results. Transferability involves how applicable the findings are to other settings. To support this, this study included detailed descriptions of the context, participants, and research procedures, helping readers judge if the findings could be relevant elsewhere. Background information on the participants and the setting was also provided. Confirmability ensures that the results are based on the participants’ input rather than researcher bias. An audit trail documented decisions and research steps, while peer debriefing allowed colleagues to review and provide objective feedback on the findings. Finally, dependability refers to the stability and consistency of the research over time. Thorough documentation of the process, including audit trails, was maintained. Code–recode strategies were used to verify consistency in data coding, and regular team meetings helped align interpretations and decisions.

## 3. Results

### 3.1. Sociodemographic Characteristics of the Sample

The sample was comprised of 31 CMNs, with 24 women. The participants were divided into three age groups based on age: from 40 to 50 years: 5 CMNs, from 51 to 60 years: 18 CMNs, and over 60 years: 8 CMNs. The participants’ mean age was 56.3 years (SD: 5.33; range: 46–65). As for work experience, 12 CMNs had more than 20 years of experience, 10 had between 10 and 20 years, and 9 had less than 9 years of experience.

### 3.2. Analysis Categories

The three analysis categories were as follows: 1. Impact on health. 2. Impact on resources. 3. Impact on professional satisfaction. Table 1 presents the frequency of verbatim statements from the categories and subcategories.

The verbatim statements selected were identified with the number corresponding to each of the CMNs interviewed (until reaching a total of 31), the HD to which the CMNs belonged, and their gender, all between brackets and separated by underscore symbols.

### 3.3. Impact on Health

#### 3.3.1. Patients

The CMNs believed that a number of changes related to the patients’ quality of life and health had been achieved, considering this as one of their main objectives. It was necessary to implement changes and innovations in the assistance provided to attain this goal.

*It was us that promoted the idea of inventing things ourselves to improve the patients’ quality of life, which was (and still is) our main goal*.

(24_AHD_62_F)

The CMNs could also intervene in the patients’ appointments, trying to consolidate several consultations in a single day. This task of scheduling one-stop appointments for complex patients also contributed to their quality of life and health.

*… we solve problems because we can be in contact with many people, access to this and contribute to what we call “one-stops”, not going through any usual circuit but through a parallel one and improving access to the hospital or to the tests, or even for performing complementary tests*.

(18_SHD_58_M)

The impact exerted by CMNs on the patients’ health was evident, especially in terms of caring for complex chronic patients that presented instability criteria, whose cases were handled with through therapeutic education for them to be able to self-manage their care and prevent complications.

*… in the complex chronic patients we’re working with… our intervention is influential to prevent relapses, to allow for good health management, good therapy regime management*.

(6_SSHD_57_F)

The health education provided by the CMNs to patients with chronic conditions contributed to improving quality of life in the long term.

*… I’ve taught patients to do some good diabetes self-controls, control their blood pressure, control their weight, control diuresis, check the pill for when their feet are swollen, control heart failure; if you teach patients to control all this, their quality of life improves a lot*.

(19_SHD_60_F)

At the palliative care level, the CMNs intervened even before these patients evolved to a complex situation, as these subjects belonged to these professionals’ target population or service portfolio. These patients were oftentimes referred from the hospital before they showed any deterioration; it was then that the CMNs usually intervened, before a complex situation was reached.

*At the personal level, I’m really sensitized about the topic of Palliative Care, which falls into what the CMN target population would be, these patients benefit from what I do*.

(16_SHD_59_M)

Oftentimes, with no scheduled appointments, the CMNs eased patient care by enabling the patients to access them directly; this situation also impacted the patients’ health and quality of life.

*The assistance provided by a professional gives quality of life, free access without an appointment; all this really benefits the patients… and exerts an impact on the population*.

(13_SHD_57_F)

The patients found companionship in the CMNs; in addition, home-based care was facilitated, avoiding commuting to hospitals, which in turn exerted an impact on health.

*What patients need is to feel that the health service is there with them… we’re there for the patients, we’re reference professionals*.

(27_NSHD_57_F)

*They find peace of mind, they know that there’s a person there and that she/he will go tell them what to do, be with them throughout their health process*.

(1_ESHD_62_F)

*… when patients need some home treatment to get better, we do it, so as not to take them to Seville…*.

(23_AHD_43_F)

#### 3.3.2. Caregivers

The objective pursued by some CMNs with their professional performance was to improve the patients’ and families’ quality of life, especially the caregivers’ quality of life.

*… our goal is to improve quality of life, not only the patients’ but the families’ too and, ultimately, do our share in the community where we work*.

(20_SSHD_57_M)

An important aspect inherent to CMNs in relation to health was the assistance they offered to caregivers, providing closeness, companionship, and accessibility to the health system, in addition to acting as referents in case of any complications.

*… the issue of the women caregivers that doesn’t even depend on material resources but many times on accessibility, closeness, care quality and warmth; I believe that this really improves these caregivers’ quality of life and, consequently, the patients’*.

(16_SHD_59_M)

*… the caregivers themselves tell you so, their satisfaction level, how coping with the care measures and with that situation improves thanks to your intervention… our intervention is positive…*.

(6_SHD_57_F)

In turn, this impact on the caregivers’ quality of life was positive for frail and dependent patients.

*Man, I believe that, first of all and especially, there’s a group that it’s the issue of women caring for extremely dependent patients*.

(16_SHD_59_M)

### 3.4. Impact on Resources

#### 3.4.1. Computer Tools

A number of tools were under development to better record CMNs’ performance.

*… perhaps we lack tools…*.

(28_NSHD_49_F)

The impact exerted by the CMNs’ performance on the resources was not acknowledged in quantitative terms, as these expenses were not contemplated in the Clinical Management Unit (CMU) program contract.

*but we should definitely be a guide in certain things. It’s true that there are many expenses that can be imputed to our everyday performance, which is high-cost because complexity requires so*.

(11_SHD_50_F)

#### 3.4.2. Effectiveness/Efficiency of the CMNs

In most of the interviews, the CMNs acknowledged the positive impact of their performance on the health of the population they served. They performed multiple managerial tasks to achieve results directly related to health such as reducing hospital admissions and readmissions or the number of emergency visits. This resulted in improvements in the patients’ quality of life and in significant support for the caregivers.

*Well I’m, I’d be some sort of health tool for the population and the impact on the direct assistance I offer, my management interactions with other levels, with other peers to achieve health results, fewer admissions, improvements in perceived quality of life and support for the caregivers*.

(18_SHD_58_M)

*As for quality of life, we can see the impact at the health level; as case managers, we coordinate and work multidisciplinarily, then you put a mechanism to work, if you have to refer someone to rehab, if you have to refer them to other teams or to the nutrition area, etc. You then notice the positive repercussion on the patents, I’ve already seen it and I still do*.

(14_SHD_56_F)

The CMNs seemed to have an access pathway to more information than other nurses and better accessibility with no previous appointments; this was extremely beneficial for the patients, as it improved their quality of life.

*It seems that we have some sort of access pathway to more information than the rest of the Nursing staff, The assistance provided by a professional gives quality of life, free access without an appointment; all this really benefits the patients*.

(13_SHD_57_F)

*As for quality of life, we can see the impact at the health level; You then notice the positive repercussion on the patents, I’ve already seen it and I still do*.

(14_SHD_56_F)

The time availability devoted by the CMNs to complex patients also contributed to improving their quality of life. In the interviews, it was also evidenced that some people did not need to be institutionalized in nursing homes thanks to the work done by their CMN, improving care and quality of life, which translated into fewer institutionalizations. The population acknowledged these aspects.

*… for our time availability, for our training and competences, for the resources; no doubt that quality was in fact improved, easing for more complex patients that’d be institutionalised before to stay at their homes instead*.

(11_SHD_50_F)

The CMNs were a positive resource, as their intervention sometimes streamlined the patients’ hospital discharge and due coordination with the PHC team. These actions were extremely efficient and reduced health system costs.

*Man, I think it’s a very positive resource, very positive when it comes to the good things, trying to plan a complex discharge before it comes from the hospital, for example, coordinating my work with the PHC team*.

(17_SHD_55_F)

Ultimately, the impact at the level of resources was possible because the CMNs managed and provided resources that the patients needed but the health system could not offer based on their care requirements, always aiming for this management to be as effective as possible for the patients and efficient for the system.

*… is to increase accessibility to all the resources and to everything the system can offer to these complex patients with significant care and support needs, as well as guidance on this care and for the professionals too*.

(23_AHD_43_F)

#### 3.4.3. Material Resources

Many actions and services performed by the CMNs reduced or prevented complications and favored better quality of life. This can be seen in the orthoprosthetic resources (walkers or walking sticks, for example), which the CMNs managed to prevent accidental falls in frail and at-risk patients. This work reduced health system expenses, as multiple hip fractures and other fall-related complications in older adults were prevented. The CMNs’ actions evidently exerted a large impact on the patients’ quality of life and health.

*… the patient I asked the walker for keeps falling, and when the walker arrives he’ll need a chair, he already needs a surgical intervention and has lost quality of life*.

(26_NSHD_52_F)

In relation to the impact on resources, population aging and increased dependence are creating a need to increase the number of orthoprosthetic resources to ease home-based care. This is related to borrowed materials, which the CMNs could access through a platform specifically enabled for these arrangements.

*I believe that, although it’s true that I get the impression that the resources are really, really scarce. I’m not saying there are fewer, but the population that needs them increases by the minute*.

(16_SHD_59_M)

*… the impact in terms of quality-cost, sure, we borrow a whole lot of materials, we really need to ask for a lot of materials and not stay still*.

(22_AHD_49_M)

On the other hand, the CMNs managed the material resources adequately; they even recycled orthoprosthetic materials, which resulted in favorable economic management for the health system.

*… I feel nothing but pride if I can recycle 150 wheelchairs at the end of the year, I feel proud of that because I’m teaching the health system to use resources and to share as necessary*.

(26_NSHD_52_F)

### 3.5. Impact on Professional Satisfaction

We distinguished two subcategories in this broader category, namely positive professional satisfaction and negative professional satisfaction.

#### 3.5.1. Positive Professional Satisfaction

Most of the CMNs stated having high professional satisfaction levels despite the difficulties and work overload they could sometimes be subjected to in this job position.

*… I feel satisfied and that I adapt more to what’s required from a Case Manager, I’m more focused on Case Management*.

(18_SHD_58_M)

*What prevents me from leaving is the users, the reward I get from users and also from my peers*.

(31_ESHD_63_F)

This satisfaction was also partly due to the fact that they felt they served as referents for other professionals.

*… I also get satisfaction for being like some role model for the other professionals; they always see me as a person that offers them another view or way of doing things, but that’s just because they want to put me in that position: they might as well do everything themselves and not me*.

(13_SHD_57_F)

*… I really value working as a team with doctors, nurses, social workers and all the other professionals*.

(10_SHD_61_F)

The greatest satisfaction stated by the CMNs was especially due to the assistance they offered to the patients and the results they obtained through this professional activity.

*I believe that satisfaction comes from that first, from knowing that I can get close to patients that need it and that I can help them; this is the first satisfaction I see in my job*.

(16_SHD_59_M)

Solving complex situations in the patients’ homes (which sometimes prevents institutionalizing older adults) produced significant satisfaction in these professionals. These aspects were acknowledged by the population they served and increased their satisfaction level.

*I do believe that we have some important power there and the population acknowledges that; this is the satisfaction part they do recognise, when it’s really complicated cases that we’re talking about*.

(11_SHD_50_F)

The people requiring palliative care and their family members (caregivers) that were assisted by the CMNs stated that they were highly satisfied; these situations were also a reward for these professionals, who perceived them as providing personal and professional fulfilment.

*I love working with Palliative Care patients, no job can be more rewarding: the fact of being there, offering companionship, the simple detail of human warmth that I believe is Nursing’s best; we’re caregivers, the thing of you lending a hand to a caregiver and that warmth you pass on to her, that’s wonderful, grief follow-up*.

(3_SSHD_62_F)

#### 3.5.2. Negative Professional Satisfaction

In terms of negative professional satisfaction, some of the CMNs stated that they were a figure that could offer much more due to the vocational and altruist aspects inherent to the professionals holding these job positions.

*… I believe that we’re a figure with a whole lot more potential and that I’ve sometimes felt that, that we’re somewhat between vocationally and professionally trained because nobody… come on, they throw any task upon us and we delve into it*.

(30_ESHD_63_F)

*… it’s satisfactory at the professional level, but it could be a whole lot more and we could give much more*.

(5_SSHD_63_F)

On the one hand, the reasons for dissatisfaction were related to the tasks they performed that were not inherent to CMNs, and on the other hand, to the teams not knowing about their roles and competences.

*… we should insist again on clearly explaining which our roles are and, especially, what we’re here for; given our competences, we should also be some methodological and research support for the units…*.

(11_SHD_50_F)

On the other hand, the CMNs asserted that they were in charge of very broad areas due to their extensive geographical dispersion. They especially served very disperse rural areas, and this precluded properly caring for the patients and caregivers in person, sometimes with the need to contact them via telephone calls and losing this closeness and in-person assistance that both patients and caregivers require in the face of difficult and complex situations. This entire situation led to dissatisfaction related to their professional activity.

*… I’m talking to you from my area; it’s very hard for us, a lot of work, we serve a very large population. I have some geographical dispersion You do what you can and I tell you, a lot via telephone calls and, for me that I like to be close to the patients, I do most of them in home visits…*.

(29_ESHD_57_F)

*Sometimes I feel dissatisfied because I can’t give my best, even if it doesn’t depend on me*.

(2_SSHD_58_F)

## 4. Discussion

The main results show that the PHC CMNs included in this study asserted that their professional performance improves quality of life and satisfaction in complex patients (such as those requiring palliative care and their caregivers) through care coordination and various levels. This performance exerts an impact in relation to health resources, achieving more effective and efficient results for health systems [12,13,17,18,20,22,27]. However, some authors such as Stokes et al. (2015) do not endorse the case management model as an effective and efficient model, as they consider that for complex patients, the intervention of a multidisciplinary team is needed, and only then could the effectiveness of the case management model increase [28].

Some of these improvements are related to managing appointments; however, they are fundamentally due to health education centered on care self-management and on empowering the patients, planning the care to be implemented and solving health problems, especially in patients with chronic diseases. These results are in line with the results of other studies [2,3]. However, the CMNs stated that there was a lack of consensus regarding the definition, essential components, and adequate application in relation to competencies and portfolio of services. Wood et al. (2021) stated that an advanced practice nursing GC model should be defined, which focuses on a continuous process of care that integrates medical and psychosocial resources to achieve good results and allows for the participation of patients in their care with the healthcare team [24].

In relation to the patients requiring palliative care, the CMNs intervened before any unfavorable evolution arose and prior to reaching a complex situation. These services were part of these professionals’ portfolio. These patients were attended to with no need for previous appointments. The CMNs offered them counselling, support, and companionship, acting as referents in PHC [8,22,23].

The CMNs stated that the Andalusian Care Strategy [22] specifies quality of life and satisfaction among its objectives, and one of the CMNs’ roles was to assist people in palliative care.

Caregivers were part of the CMNs’ service portfolio. They offered them closeness, companionship, and accessibility to the health system. They also acted as referents for these caregivers. In this sense, Domenech et al. [17] stated that CMNs provide caregivers with emotional and instrumental support, as well as accessibility (either in-person, via telephone calls, or in the home environment), which provides significant satisfaction to patients and caregivers alike.

The interventions implemented by the CMNs reduced psychological distress and improved patient and caregiver safety, enhancing their self-control capacity. However, they believed that the effects needed to be assessed after prolonged interventions [20].

As for the impact of the CMNs’ performance in relation to the resources, they indicated the need to implement tools in the Andalusian Health System computer systems in order to improve the records related to their activity and allow the health results to be better assessed and quantified. This result coincides with Romero et al. [4], who stated that new care models that incorporate intermediate assistance are necessary, offering improvements in relation to care continuity and to interprofessional and interlevel communication. In addition, it would be important to incorporate Telenursing and follow-up after hospital discharge from PHC to improve the patients’ quality of life [14,15,29,30].

The CMNs stated that if their professional activity was included in the Andalusian Health Service program contract, the results would be more visible and be part of the PHC centers’ objectives [31]. The CMNs recognized the impact of their activity at the level of health resources, achieving results that improved the patients’ health and quality of life. This is in line with authors such as Bamforth et al. [12] and Valcárcel et al. [13], as they reduced hospital admissions and readmissions and the number of emergency visits via effective and efficient actions, with translated into lower health expenses.

Among other reasons, the assistance provided by the CMNs produced effective results because there were no time limitations on their professional performance or on the care they provided both to the patients and their caregivers, sometimes even managing to prevent the patients’ institutionalization, thus improving home-based care [3,12,17,19,20].

At the level of material resources, the CMNs assessed the patients and their environment in a comprehensive way, providing material resources that improved health and quality of life (orthoprosthetic walking sticks, walkers, and wheelchairs, for example) to prevent the risk of falls or other complications and ease home-based care. These actions and interventions provide improvements in the patients’ health and, in turn, reduce health expenses [16,22,23].

Some authors consider that many patients remain in follow-up by the CMNs due to their complexity, and this prolongs the care provided by these professionals. However, they continue to require multiple emergency department visits and hospital admissions despite this follow-up; therefore, it is necessary to improve the identification of complex chronic patients who require care by CMNs [32].

In relation to the orthoprosthetic materials (wheelchairs, walkers, and walking sticks, for example), the CMNs were in charge of recycling them, with positive results for the health systems because their expenses were lowered [8,16].

In coordination with the hospital and PHC, the CMNs managed the patients’ discharge. They reduced hospitalization times by streamlining discharge paperwork and management so that the patients could return home as soon as possible and be offered the best care measures. In turn, this had repercussions in the impact at the level of resources and improvements in the patients’ and caregivers’ quality of life [16,22,23].

The CMNs expressed great satisfaction for the care they provided to their patients and caregivers, especially in the case of complex patients or those in need of palliative care. This was because they provided effective and efficient assistance through interprofessional and interlevel coordinated actions, thus ensuring compliance with the therapeutic plan [18,22].

In turn, both the patients and their caregivers also expressed significant satisfaction toward the assistance received from the CMNs, feeling more accompanied and supported at the emotional and instrumental levels [17,20,30]. Further research studies are needed, according to Sadler et al. (2023), to determine whether CMNs’ interventions benefit certain individuals [33].

The CMNs acted as leaders or referents in PHC teams. However, they mentioned the importance of the other team members knowing their roles and competences. This way, the CMNs might better develop their professional activity, and their satisfaction level would be enhanced. In turn, this would improve the health results [9,11,14,15]. However, authors such as Putra and Sandhi (2021) consider that there is a need to improve the continuing education of CMNs in order to achieve better competencies and results [34].

Some authors consider that in order to achieve these positive results of CMNs, evidence-based practice is needed, and for them, a common theoretical model that unifies the practices of these nurses at an international level is needed [32].

A major drawback is that institutional guidelines have been lost; something that Casado et al. (2017) agree with. This manifests itself as a loss of the institutional support that existed at its origins in 2002. This was expressed in the interviews as a negative aspect, as there was no longer a common and homogeneous policy regarding the implementation of these positions [16].

Authors such as Morales et al. state that there is enormous variability among these professionals (CMNs), so larger studies with longer follow-ups are needed to generalize the data at an international level [32].

Among the strengths of this study, we can highlight that it followed a qualitative methodology focused on in-depth interviews, which allowed the CMNs to express their opinions, sensations, and perceptions with no time restrictions by scheduling dates and times when they were available.

In relation to the limitations of this study, we can note the fact that 31 CMNs from the different Health Districts in the province of Seville were interviewed following convenience sampling, which may have excluded other CMNs working in other HDs and provinces who might have other opinions or perceptions.

Authors such as Joo and Liu comment that recent qualitative studies regarding the experience of case management in people with chronic diseases and their caregivers are needed in order to generalize data at an international level [35].

This research is important to clinical practice because if systems to assess CMNs’ performance were implemented, the results of their professional activity would be made visible, demonstrating the effectiveness and efficiency inherent to these professional profiles in relation to the assistance provided to patients with complex chronic conditions and their caregivers. Likewise, future research is required to demonstrate their effectiveness/efficiency in relation to the quality of care and the reduction of clinical variability [16,18].

## 5. Conclusions

Health models should adapt to the new requirements derived from population aging, increased life expectancy, and higher numbers of people with chronic health problems. This requires interventions by CMNs to attain results that improve health, quality of life, and satisfaction level in patients and caregivers alike, with a need for proper management of health resources. Hence, it is important to have tools that allow for recognition and assessment of CMNs’ professional activities, in addition to the need to implement this figure at the international level.

The CMNs reported high levels of job satisfaction. The CMN Advanced Practice care model is fundamental to improving the assistance provided, both at the home level and in nursing homes. Nevertheless, more profound implementation of these advanced practice models is required, as promoted by the WHO and the PAHO. In addition, new research studies that assess advanced practice competences and confirm the results and their generalization are required.

## Figures and Tables

**Table 1:** Categories, subcategories, and frequency of verbatim statements.

Category	Subcategories	Frequency of Verbatim Statements
Impact on health	Patients	28
	Caregivers	13
Impact on resources	Computer tools	4
	Effectiveness/efficiency of the CMNs	65
	Material resources	9
Impact on professional satisfaction	Positive professional satisfaction	157
	Negative professional satisfaction	98
		374

## Data Availability

No additional data are available.

## Abbreviations

The following abbreviations are used in this manuscript:

CMN	Case-Management Nurse
PHC	Primary Health Care
SRQR	Standards for Reporting Qualitative Research
WHO	World Health Organisation
PAHO	Pan-American Health Organisation
CM	Case Management
HD	Health District
AHD	Aljarafe Health District
ESHD	East Seville Health District
NSHD	North Seville Health District
SHD	Seville Health District
SSHD	South Seville Health District
